# Advancing clinical translation of cardiac biomechanics models: a comprehensive review, applications and future pathways

**DOI:** 10.3389/fphy.2023.1306210

**Published:** 2023-11-14

**Authors:** Cristobal Rodero, Tiffany M. G. Baptiste, Rosie K. Barrows, Alexandre Lewalle, Steven A. Niederer, Marina Strocchi

**Affiliations:** 1 Cardiac Electro-Mechanics Research Group (CEMRG), National Heart and Lung Institute, Faculty of Medicine, Imperial College London, London, United Kingdom; 2 Department of Biomedical Engineering, School of Biomedical Engineering and Imaging Sciences, Faculty of Life Sciences and Medicine, King’s College London, London, United Kingdom; 3 Turing Research and Innovation Cluster in Digital Twins (TRIC: DT), The Alan Turing Institute, London, United Kingdom

**Keywords:** mechanics, biomechanics, electromechanics, electro-mechanics, cardiac model, cardiac simulation, heart model, heart simulation

## Abstract

Cardiac mechanics models are developed to represent a high level of detail, including refined anatomies, accurate cell mechanics models, and platforms to link microscale physiology to whole-organ function. However, cardiac biomechanics models still have limited clinical translation. In this review, we provide a picture of cardiac mechanics models, focusing on their clinical translation. We review the main experimental and clinical data used in cardiac models, as well as the steps followed in the literature to generate anatomical meshes ready for simulations. We describe the main models in active and passive mechanics and the different lumped parameter models to represent the circulatory system. Lastly, we provide a summary of the state-of-the-art in terms of ventricular, atrial, and four-chamber cardiac biomechanics models. We discuss the steps that may facilitate clinical translation of the biomechanics models we describe. A well-established software to simulate cardiac biomechanics is lacking, with all available platforms involving different levels of documentation, learning curves, accessibility, and cost. Furthermore, there is no regulatory framework that clearly outlines the verification and validation requirements a model has to satisfy in order to be reliably used in applications. Finally, better integration with increasingly rich clinical and/or experimental datasets as well as machine learning techniques to reduce computational costs might increase model reliability at feasible resources. Cardiac biomechanics models provide excellent opportunities to be integrated into clinical workflows, but more refinement and careful validation against clinical data are needed to improve their credibility. In addition, in each context of use, model complexity must be balanced with the associated high computational cost of running these models.

## 1 Introduction

### 1.1 Cardiac physiology and pathophysiology

Cardiac biomechanics consists of the study of one or more of the processes involved in cardiac function. By studying the biomechanics of a patient’s heart and how it is different depending on the patient’s demographics and health state, we can optimise therapies, help planning procedures, or learn about pathophysiology. Historically, this study has been conducted clinically and experimentally, but more recently computer models have helped in this task [1]. Computational biomechanical modelling tools can aid in this effort, providing information that is inaccessible, too invasive, or unethical to obtain clinically. These tools must also be sufficiently robust to work with typically sparse clinical data and their associated uncertainty. Mathematical representations of fundamental mechanisms can provide the basis for a bottom-up reconstitution of whole-heart function *in silico*. Using this framework, cellular mechanisms such as ion exchange and cellular force generation can be linked to changes in the macroscopic behaviour, affecting clinical, measurable biomarkers such as ejection fraction.

**Figure 1:**
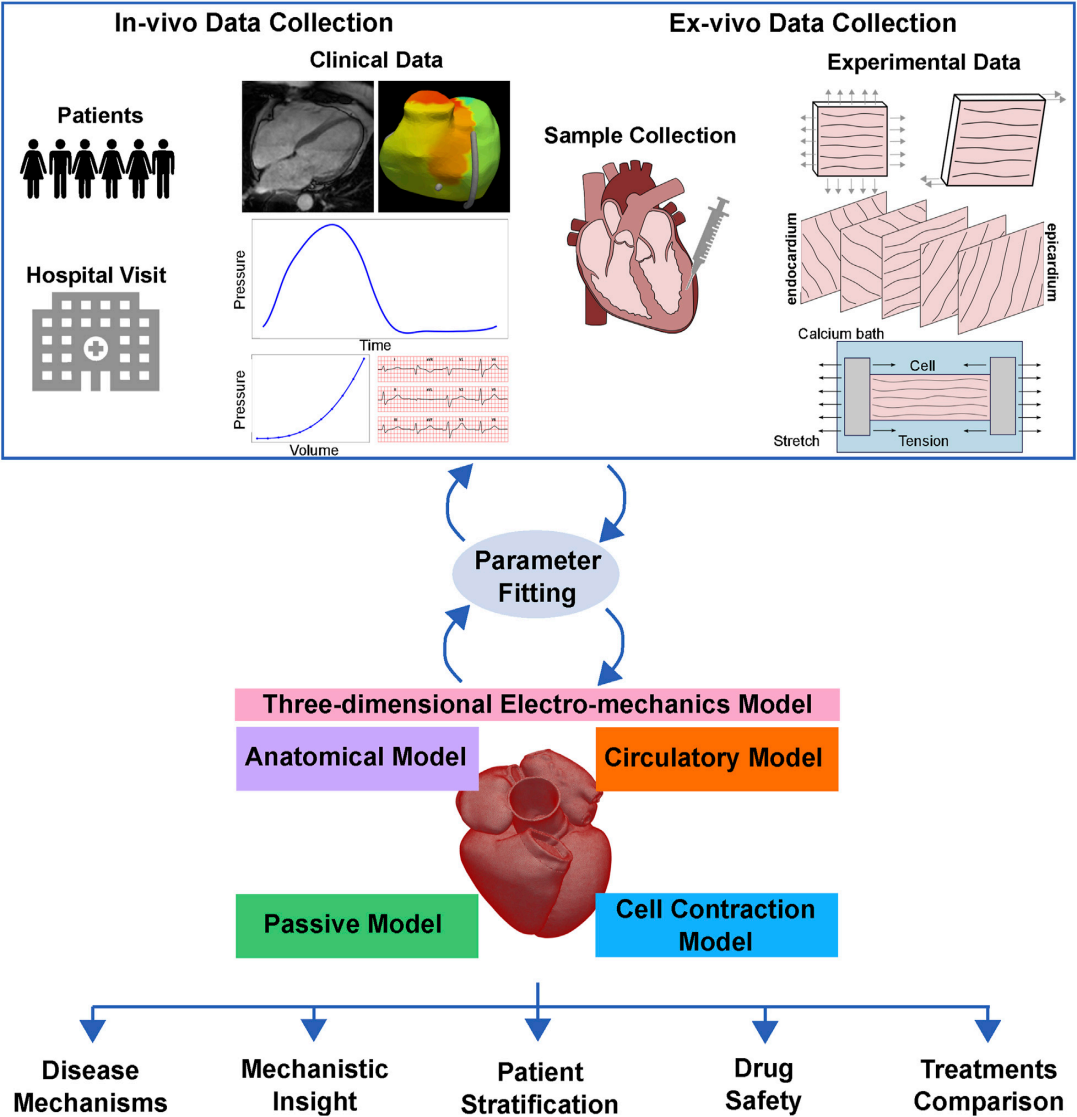
Clinical translation of cardiac biomechanics models. The clinical application dictates the type of cardiac biomechanics model to employ: passive, circulatory system, cell or three-dimensional multiscale model. *In-vivo* clinical data collected from patients or *ex-vivo* experimental data are used to fit the model parameters and/or to validate model predictions. Once validated, the model can be used to provide mechanistic insight into processes underlying cardiac function, understand disease mechanisms, perform patient stratification, and test and compare different treatments.

In Figure 1, we outline the coupling between a typical clinical and experimental pipeline with the computationally modelling approach. Briefly, the clinical application dictates the type of cardiac biomechanics model to employ: passive, circulatory system, cell or three-dimensional electromechanics multiscale model. *In-vivo* clinical data collected from patients (top left of Figure 1) or *ex-vivo* experimental data (top right of Figure 1) are used to fit the model parameters and/or to validate model predictions. Once validated, the model can be used to provide mechanistic insight into processes underlying cardiac function, understand disease mechanisms, perform patient stratification, and test and/or compare different treatments (bottom of Figure 1). The continuous development and refinement of cardiac mechanical models witnessed over the past half century, together with increase in computational power, make this goal increasingly realistic.

In this review, a mechanics model refers to a mathematical or computational representation of the physical properties and behaviours of cardiac tissues, cells (cell models), or the circulatory system (circulatory system models). To improve readability, when not explicitly specified (for example, with the term ‘whole-heart modelling’), we mean both anatomical and functional modelling. When only the geometry is replicated, we use the term ‘anatomical model’. We use the term ‘simulation’ to denote the computational processes that aim to replicate cardiac biomechanical phenomena. Unless stated otherwise, when we refer to techniques such as parameter estimation, it refers to *in-vivo* parameter estimation for patient-specific parameters, typically via inverse modelling.

### 1.2 Previous reviews of cardiac mechanics computational models

Cardiac mechanics models are developed to represent a high level of detail, including refined anatomies, accurate cell mechanics models, and to account for mechanisms of interaction between different chambers such as the pericardium, and the circulatory system, and between different physics through mechanoelectric feedback [1]. In recent years, multiple reviews have been published on cardiac biomechanical modelling. In [2], Wang et al. discussed some of the main articles on cardiac biomechanical models, with an emphasis on the images from which the anatomical data were extracted (for an introductory overview of image-based anatomical and mechanical modelling, see [3]). The focus was mainly on (left) ventricular properties and mechanics modelling, while mentioning several studies in which left ventricle (LV) modelling helped improve our understanding of the mechanisms underlying cardiac diseases. In 2019, Avazmohammadi et al. [4] summarised the last 30 years of myocardial biomechanics modelling focusing on constitutive equations, highlighting the need for better integration between clinical and experimental data with computational methods. This integration was the focus of the review by Bracamonte et al. [5], that is, on patient-specific inverse modelling. Several authors have summarised the main challenges of modelling cardiovascular mechanics, restricting their reviews to specific aspects of cardiac biomechanics, such as multiscale modelling [6] or computational models of specific pathologies [7–9].

Models of cardiac mechanics have been formulated for almost a century [1]. However, only recently did these models start to generate compelling evidence of their clinical relevance, paving the way for their clinical translation. In [10], Lesage et al. conducted a survey asking 163 clinicians about their familiarity with modelling and found that computational modelling-related terms such as patient-specific modelling are becoming well known. More than half of the responders had already used computer modelling and simulations to plan procedures, 57.4% of them in the cardiovascular field. Despite some potential sampling bias, this study indicates that clinical translation of cardiac computational modelling is already happening. However, none of the reviews mentioned above provided a summary of the state-of-the-art of the clinical translation of cardiac mechanics models.

In this review, we provide a picture of some of the main developments towards the clinical translation of cardiac mechanics models. In Section 2, we describe the main clinical and experimental data and methods used by the anatomical models and the simulations. In Section 3, we describe the processes of anatomical model generation and the incorporation of the fibre orientation and scar. We then describe the state-of-the-art of cellular contraction models in Section 4 and passive mechanics in Section 5. We review circulatory system models in Section 6 and ventricular, atrial, and four-chamber electromechanics models in Section 7. In Section 8 we briefly discuss how machine learning is influencing model translation. Lastly, in Section 9 we discuss the clinical applications of all the discussed models and the challenges that currently hinder the clinical translation of cardiac mechanics models. The final remarks of this review are given in Section 10.

## 2 Experimental and clinical data used in anatomical and mechanics models

There is a plethora of clinical tools and equipment available for collecting a wide range of data from a patient’s heart, including detailed anatomical, structural, and functional data. However, even given all this information, the clinical decision-making process remains challenging, as there is no tool capable of combining all this information into a unified framework. Computational electromechanics models have the potential to fulfil this purpose by unifying all clinical information into a multiscale, multiphysics and physics-constrained platform. In this section, we summarise the clinical and experimental data available to modellers to constrain and validate electromechanics models.

### 2.1 Anatomical and structure measurements

Anatomical information can be recovered from *in-vivo* or *ex-vivo* data. Histology can be used to obtain slices of hearts from different species, obtaining sparse shape information. Explanted hearts can also be imaged to obtain high-resolution images of the heart and surrounding tissues. However, *ex-vivo* imaging cannot be used for longitudinal or diagnostic studies. *In-vivo* imaging, on the other hand, allows one to collect longitudinal data for the same patients. Both *ex-vivo* and *in-vivo* hearts can be imaged by techniques such as computed tomography (CT) or magnetic resonance imaging (MRI). In contrast to CT, MRI does not require any ionising radiation and can therefore be used to image healthy subjects for multiple acquisitions over a short period of time while avoiding radiation exposure. However, MRI requires long acquisition times, which requires multiple breath holds from the patients and may be difficult for patients with cardiac disease. Moreover, it is not always suitable for patients with devices implanted and has low long-axis resolution. In these cases, CT provides submillimetre isotropic resolution, but involves ionising radiation. Echocardiography provides a rapid and inexpensive option to assess basic anatomical measurement using ultrasounds at the expense of accuracy.

Diffusion tensor MRI (DT-MRI) and histology can also be used to recover information about cell alignment within the myocardium. In the heart, myocytes are organised into fibre bundles. Although histology measurements are often used as a ground truth for the estimation of fibre direction [11], they are sparse and time consuming and are associated with an uncertainty of about 10° [11]. DT-MRI is a technique that allows to map the predominant direction of water molecules diffusion within the tissue, providing essential insights into the underlying fibre structures. When applied *ex-vivo*, this provides high-resolution data about fibre distribution in the whole heart. Although *in-vivo* DT-MRI is limited by cardiac motion and long acquisition times, it has been used to detect fibre disarray and remodelling in patients with hypertrophic [12] and dilated [13] cardiomyopathies, showing its potential for future clinical applications.

Scarring and fibrosis occur when cardiac tissue dies due to a lack of blood supply. Scar tissue constitutes a substrate for a wide range of pathologies, such as atrial fibrillation (AF) or ventricular tachycardia, and its distribution is used to identify potential ablation targets to prevent life-threatening arrhythmias. This makes scar estimation in cardiac patients crucial. Late gadolinium enhancement MRI can be used to quantify the extent, transmurality, and mass of the myocardial scar, which appears as a bright area on cardiac images. However, late gadolinium enhancement techniques are currently unable to detect diffuse myocardial fibrosis, which is an earlier form of fibrosis preceding replacement fibrosis that may be reversible. T1 mapping refers to information derived from MRI images to measure a mathematical constant, called T1 relaxation time. The main advantage over late gadolinium enhancement is that T1 mapping MRI does not require contrast and allows a quantitative measurement of features such as diffuse fibrosis, a reason why it has recently been used to detect this type of fibrosis [14]. Scar can also be quantified with electroanatomical mapping, as low-voltage areas have been shown to correlate with scar in both ventricles [15] and atria [16], although transmurality information is limited. Finally, the quantification of thin areas of the ventricular wall on CT with delayed enhancement has been correlated with areas with low voltage on electroanatomical mapping, but this is not widely used in clinical practise to identify scar tissue [17]. For a more thorough review on the link between anatomical and functional modelling and imaging, we refer the reader to [3].

2.2 Tissue and cell measurements

Passive tissue measurements have advanced our understanding of the mechanical properties of the myocardium. Initial evaluation of passive properties of the myocardium was carried out through uniaxial tests (e.g., stretching the sample in the longitudinal direction) in rabbit papillary muscle samples, showing the nonlinear behaviour of cardiac tissue [18]. Later, biaxial tests on canine myocardial strips excised from the subepicardium of the LV free wall, carried out by stretching the sample in the fibre and cross-fibre directions, showed increased stiffness in the fibre direction, indicating that the myocardium behaves as a transversely isotropic material rather than an isotropic material [19, 20]. Finally, more complex shear tests were used to investigate the shear properties of the myocardium. Using these types of measurements on tissue samples from the LV lateral wall, Dokos et al. [21] concluded that cardiac tissue is actually orthotropic. Most tissue measurements are performed on ventricular samples, leaving the properties of the atrial tissue poorly characterised. Bellini et al. [22] provided the first and only experimental dataset that characterises the passive properties of the healthy human myocardium of the left atrium (LA) and the right atrium (RA) by performing biaxial tests on samples collected from five different areas of the atria.

A substantial proportion of experiments investigating the active properties of the cardiac muscle are carried out in skinned muscle preparations, where the sarcolemmal membrane has been dissolved [23]. The benefits of these experiments include 1) the ability to tailor the biochemical environment of the sarcomere components; 2) the isolation of the contraction mechanisms from other cellular subsystems, such as the sarcolemmal membrane; and 3) the ability to preserve the system for several months (particularly useful for human samples, which are naturally scarce) [24]. The underlying assumption of skinned muscle measurements is that the structural and molecular features of the myofilament system are sufficiently preserved to reproduce the essential functional properties and behaviour of the original system. However, in reality, there are notable discrepancies between the skinned and intact systems, which make the translation of the measurements of the skinned system into their physiological context challenging [25]. Nonetheless, skinned muscle preparations are widely used to provide quantitative information about active tension properties and cardiac cell kinetics.

Force-calcium (F-Ca) relationships provide a conventional basic characterisation of steady-state force generation at the sarcomere level [26]. Typically measured using skinned preparations (although they have also been measured in intact myocytes [27]), the F-Ca relationships are used to measure the force generated by muscle fibres at different levels of calcium concentration and under different conditions, for example, sarcomere length [28]. More precise measurements have identified an asymmetry in the F-Ca curves [29].

More complex experiments carried out on both skinned and intact muscle preparations have been used to provide in-depth information about the system kinetics and to quantify the force generated by cellular contraction. Dominant processes can be observed by applying small length perturbations [28] or by measuring dynamic stiffness through a strain of varying frequency [30]. These experiments provide an intuitive framework for interpreting kinetics-modifying interventions, such as the application of the contraction inhibitor mavacamten [31]. Tension-recovery experiments, also performed in skinned preparations, probe the cross-bridge turnover dynamics. These experiments involve rapidly slackening the muscle fibre to zero tension, followed by rapid restretching, assumed to cause cross-bridge dissociation [32]. Therefore, the observed tension recovery reflects the dynamic reforming of the cross-bridges. Controlling calcium concentrations precisely in skinned muscle preparations on short time scales poses a challenge. Consequently, experiments that measure muscle twitches are conducted on intact cells, rather than skinned muscle preparations, to study dynamic force generation in response to calcium transients [33]. The experimental data described above are based on complex procedures and setups, making data collection time consuming and challenging. Furthermore, these experiments allow one to observe only very specific processes and, therefore, lack the link to other mechanisms affecting cardiac function as a whole.

### 2.3 Organ-scale measurements

Measurements carried out in myocardial tissue samples are fundamental to investigate tissue properties, both active (e.g., force generation), and passive, (e.g., material stiffness). However, in some cases, *in-vivo* measurements might be needed instead. In this section, we provide a brief summary of the main types of data and their collection techniques used to validate mechanics simulations.

Time-volume traces can be non-invasively derived from cine MRI. However, in some cases, MRI is not indicated, for example, due to the presence of a device. In such cases, if ionising radiation is not a concern, time-volume traces can be derived from retrospective gated cardiac CT. Cine MRI suffers from lower long-axis resolution compared to CT, but retrospective gated cardiac CT has a poorer temporal resolution than cine MRI. Echocardiography constitutes a non-invasive, inexpensive and widely available measurement of volumes, ejection fraction, and local longitudinal strains for all cardiac chambers. Tagged MRI and retrospective gated cardiac CT can also be used to measure local strains, but interpreting these data requires more advanced analysis, such as motion tracking and deep learning.

In addition to imaging, cardiac biomechanics can be characterised clinically using a range of diagnostic measurements. *In-vivo* end-diastolic pressure-volume relationships (EDPVRs) can be invasively measured using pressure-conductance catheters, providing simultaneous recordings of pressure and chamber volumes [34]. Invasive catheter measurements of LV pressure over time can be used to quantify the efficiency of myocardial contraction and relaxation using peak pressure and maximum and minimum pressure time derivatives. For cases where time traces of volume and/or pressure measurements are not available, [35] proposed a law (the so-called Klotz curve) to estimate EDPVR for a specific patient based on a single pressure-volume measurement. Clinically, EDPVR is used to assess the passive mechanical behaviour of a patient’s heart and can be used to identify the presence of fibrosis, remodelling, or hypertrophy [36].

Although they do not provide direct information on cardiac function, electrical measurements are widely used in the clinic in combination with functional mechanical measurements described above, and can be used to validate the electrical activation simulated in a multi-scale electromechanics model. Twelve-lead electrocardiograms (ECGs) are routinely used to identify myocardial scar, dyssynchrony, and other electrical disturbances. Body surface potential maps and electrocardiographic imaging are an extension of standard ECGs, with more recording electrodes located on the patient’s torso, and can provide more detailed information about the electrical activity of the heart. However, these techniques are not intended for routine clinical use and are restricted to specific use cases, such as response to cardiac resynchronisation therapy (CRT) [37, 38] or different types of arrhythmias [39].

All the data mentioned above can be used to validate the heart’s mechanical behaviour simulated by different types of computational biomechanics models of the heart. In the following section, we describe how anatomical models of the heart are built, mainly using three-dimensional imaging techniques such as CT or MRI.

## 3 Anatomical model generation

**Figure 2:**
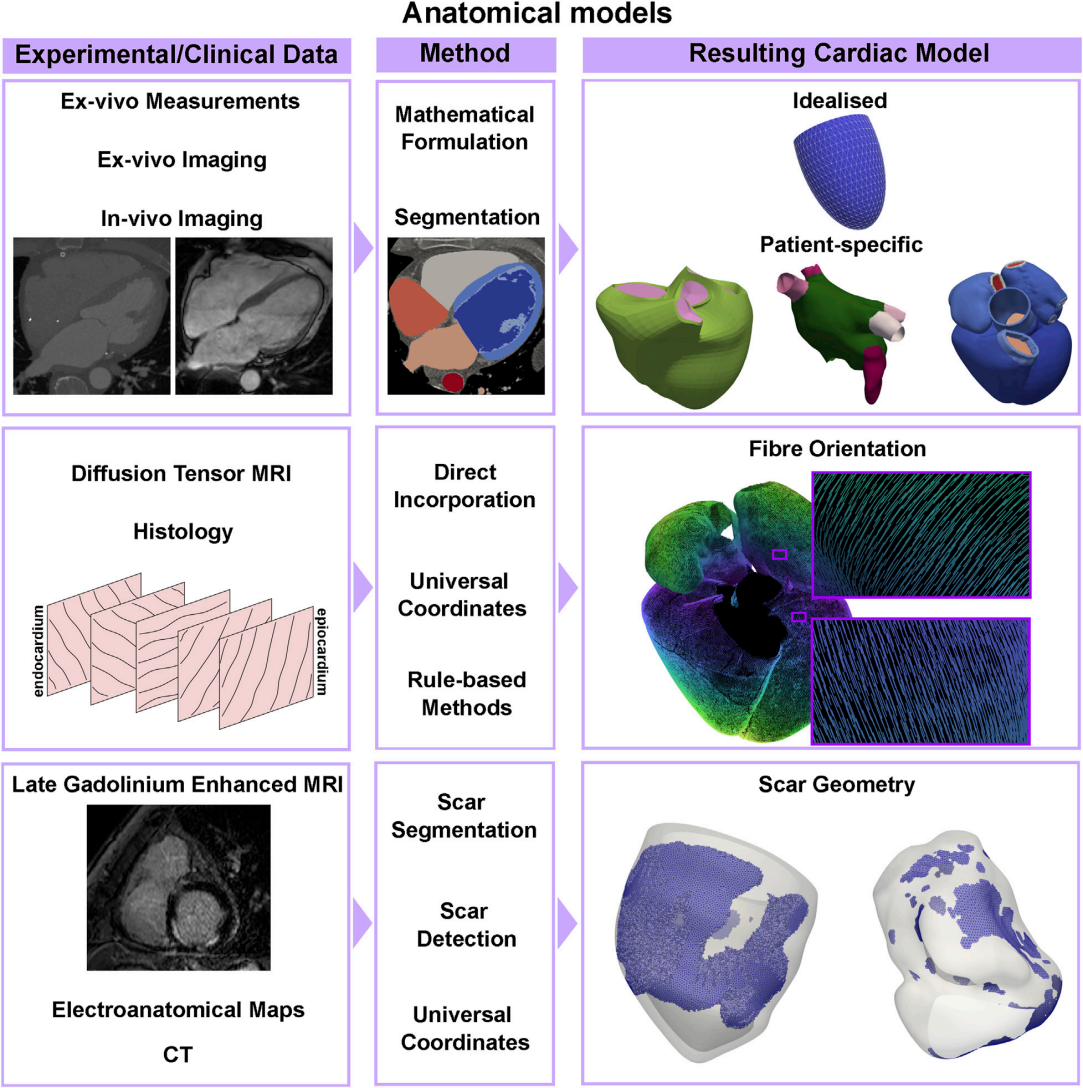
Anatomical models pipeline. An idealised geometry can be built using measurements from *ex-vivo* hearts and a mathematical formulation for a truncated ellipsoid, while patient-specific anatomies of ventricles, the atria, or the whole heart can be segmented from CT or MRI imaging data. Atrial and ventricular fibre orientations can be directly incorporated or mapped from *ex-vivo* DT-MRI datasets, or by using rule-based methods based on sparse histology data. Scarred tissue can be detected using late gadolinium enhanced MRI, thresholding electroanatomical voltage maps or by applying machine learning methods to CT datasets.

The first animal [40] and human [41] anatomical models of the heart were based on the LV, partially due to its regular shape, well approximated by a truncated ellipsoid. Increased availability of experimental data have made the realisation of more detailed anatomical models possible. Biventricular anatomical models of mammals have been generated using digital images of short-axis histological slices [42], *ex-vivo* MRI images of histological samples [43–45], and *ex-vivo* whole heart MRI [46–48]. The growing interest in the effect of the atria on cardiac function has led to increasingly anatomically detailed models generated from histological slices of the sheep atria [49], the human atria [50, 51] and the whole human heart [52]. Advances in *in-vivo* imaging allow one to acquire highly detailed anatomical datasets. Both MRI and CT have been used to generate patient-specific anatomical models of the ventricles [53–57], the LA [58–60], the atria [61], and the whole heart [56, 62, 63], as shown in the first row of Figure 2.

Fibre orientation in the ventricles has been directly incorporated from high-resolution *ex-vivo* DT-MRI datasets [43, 46, 64–66] or by registering histological data in a computational geometry [42], see the second row, left, of Figure 2. Although these methods provide measurement-based fibre architectures, they can be considerably difficult to apply *in-vivo*. For this reason, rule-based methods [67] remain popular. Essentially, these methods establish a series of rules for describing fibres in the myocardium based on (typically canine) histological data. Several rule-based methods have been developed for the ventricles [68–73] and atria [58, 71, 73–79]. Ventricular fibres can also be mapped from a geometry with a known fibre orientation using universal ventricular coordinates [80], a set of coordinates that uniquely define a point within a biventricular anatomy independently of the geometry. Similarly, universal atrial coordinates have been used to map *ex-vivo* DT-MRI fibres from an atlas onto patient-specific biatrial anatomies [59, 60, 81]. An example is shown in the right side of the second row of Figure 2. Due to the challenges of acquiring high-resolution *in-vivo* DT-MRI datasets, patient-specific fibre orientation is not yet achievable in cardiac anatomical models.

Depending on the application, tissue heterogeneity might play a role in the dynamics of interest. For example, accounting for the scar and border zone (the region usually surrounding the scar with abnormal conduction properties) in the anatomical and mechanics model is important when trying to detect the optimal location for leads in pacemaker implantation, to understand the strain and work distributions in different pathological conditions, or to reproduce reentrant waves during arrhythmias. More recently, machine learning techniques have been used to identify scar areas on delayed enhancement CT imaging [82] and CT angiography [83]. Electroanatomical voltage mapping can also be used to detect fibrotic areas by thresholding the voltage in the atria [84, 85] and in the ventricles [86, 87] to detect low-voltage areas, which can be surrogates for the scar. The typical data and methods can be seen in the bottom row of Figure 2, as well as an example of scar geometry in two ventricular meshes. However, in the ventricles, it can be hard to discern whether this scar is endocardial or transmural. When clinical data are not available, universal atrial and ventricular coordinates can also be used to map segmented scar areas from one geometry to another.

Once the anatomical model is built, functional properties must be assigned to the tissue. These can be classified as active or passive depending on whether the tissue generates force or not, respectively. In the following sections, we summarise the state-of-the-art for the active mechanics at the cellular level and the passive mechanics at the tissue level.

## 4 Active cell mechanics

Single-cell experiments have provided essential insight into myocyte biomechanics. However, these experiments typically focus on measuring specific effects in isolation and do not always capture the interactions between different mechanisms that occur simultaneously. In this context, the biomechanical modelling of cell contraction potentially provides a theoretical framework for integrating experimental measurements into a unified picture [4, 88].

**Figure 3:**
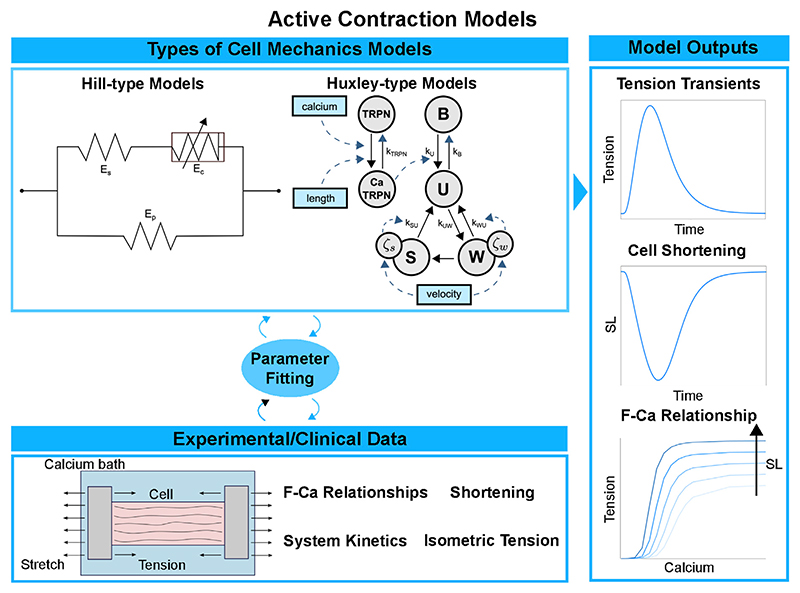
Cell contraction models pipeline. Simplified cell models directly computing the active tension transient can be used to save computational cost and discard the kinetic processes underlying active force generation. More complex cell models explicitly represent the molecular processes of the cross-bridge cycle, discretising it into a finite number of states. Different types of experimental data are used to fit the parameters and validate the cell model outputs: isometric tension transients, cell shortening and F-Ca relationships under different sarcomere length conditions.

Various paradigms of biomechanical models of force generation at the cell level have been proposed over the years. A depiction of the main ones can be seen in Figure 3. The Hill model of 1938 [89] was designed to reproduce careful measurements of the relationship between force and muscle velocity on a phenomenological basis, without invoking specific mechanisms of force production within the muscle. This simplified representation reduces the computational load and facilitates large multiscale analysis pipelines [53]. These multiscale pipelines include the integration of cardiac anatomy, electrophysiology, biomechanics, and hemodynamics in a patient-specific model of heart failure (HF) [55, 90]. In this context, the phenomenological nature of the cell model may well suffice in some data analysis pipelines designed for therapy planning, e.g., personalised cardiac CRT in a large cohort of patients [90].

In contrast, the Huxley model of 1957 [91, 92] sought to reproduce muscular function by starting from a more explicit representation of molecular interactions at the sarcomere level. In this framework, the force is generated from the strain of an elastic “spring” associated with the bound cross-bridge. Many subsequent Huxley-inspired models vary in their degree of sophistication. The cross-bridge cycle is represented in terms of transitions within a kinetic system comprising two [93], three [94, 95], four [28, 96], or more (e.g., [97–99]) states. In the hybrid approach by Gusseva et al. [100], a Hill-type model is used where the active behaviour of the myocardium is modelled within Huxley’s filament theory. In general, the computational cost increases with complexity and is typically much higher with Huxley-type models compared to Hill-type models. Force predictions in cellular biomechanical models are typically validated using measurements of F-Ca relationships, tension-recovery curves, or dynamic stiffness measurements and twitch dynamics in intact fibres, as seen in the bottom and on the right of Figure 3. *In-vitro* solution-kinetic data may also provide kinetic rate estimates of specific state transitions in contraction models. Constraining parameter values in complex mechanics models inevitably involves some degree of “inheritance”, that is, using experimental data from disparate existing sources that were sometimes obtained under inconsistent measurement conditions, which constitutes a source of uncertainty.

Alternative modelling approaches use a more abstract perspective based on the mass-action principle. They express biochemical transitions as “probability fluxes” between system states that may formally amalgamate ensembles of actual molecular states. Essential experimentally observed dynamic features can be reproduced, often requiring fewer *ad hoc* assumptions [101, 102]. The relative simplicity of such cell models arguably facilitates parameter calibration using more limited and more consistent datasets, unlike recent state-of-the-art platforms for simulating contraction [98, 103–105] that rely on calibrations derived from a wide set of experimental sources.

Spatially explicit cell models can extend the investigation of cross-bridge dynamics to filament interactions on the scale of the half-sarcomere. Fenwick et al. [106] suggested a mechanistic explanation of the sarcomere length dependence of force production. The status and location of individual myosin heads and actin binding sites can also be dynamically tracked to allow the exploration of the biophysical interactions of sarcomere components [103]. Cell models with resolution at the protein level provide a framework to investigate drug action pathways [107]. In particular, genetic mutations with a role in cardiomyopathies can serve as therapeutic targets [108]. Such effects, in principle, can at best be included only phenomenologically in spatially implicit cell models.

Modelling can provide insight into the impact of genetic variants of sarcomeric proteins and their manifestation under pathological conditions. In [109], the authors constructed an electromechanical cardiomyocyte model to reproduce phenotypes in hypertrophic cardiomyopathy, linking alterations in model parameters with mutations in myosin heavy chain and troponin genes. When the cell model is coupled with *in-vitro* measurements, they carried out an *in silico* clinical trial of the drug mavacamten, a contraction inhibitor used to treat hypertrophic cardiomyopathy. Similarly, in [105], Tomasevic et al. projected single-cell contraction onto the organ level using a finite element simulation to estimate the impact of drug interaction within cardiac cells. Therefore, this approach provides a clinical tool for tailoring drug administration to patients and altering myocardial contraction.

Coupling simulations with experimental measurements can help to elucidate the mechanism of action of drugs. Omecamtiv mecarbil (OM) was designed to directly target the cell contraction mechanism by selectively accelerating the formation of the force-generating state of the cross-bridges [110]. At the whole-heart level, it increases the duration of blood ejection without changing the contraction rate [111]. To improve understanding of the mechanism of action of OM, in [112] the authors combined a biomechanical model with machine learning to infer the model parameters from experimental data from rats. Recent modelling studies have further investigated the action of OM by simulating its impact on failing muscle. Using a cell model within a three-dimensional finite element analysis, in [113], van Herck et al. confirmed an increase in myocardial contraction driven by OM in the failing system. However, other discrepancies with experimental measurements highlight the need to further refine the cell model to fully understand the drug’s mechanism. Using Bayesian inference methods, in [114], Longobardi et al. fitted a cellular biomechanical model to *in-vivo* and *in-vitro* measurements of OM in rats. Combined with purely clinical studies of the drug, the model can assist in the development and improvement of clinical treatment.

Only myocytes activated by an action potential wave (a change in electric charge due to the exchange of ions) generate force in that instant. Nonactive tissue deforms as a result of passive forces. In the following section, we describe the main mechanics models that define how tissue deforms depending on its anisotropy and (hyper) elastic properties.

## 5 Passive mechanics

The passive behaviour of the myocardium is typically represented with a hyperelastic formulation, where stresses within the tissue are represented by a strain energy function [115]. The first material laws were developed for the LV myocardium, based on samples collected from the canine LV free wall [20]. Initially, the myocardium was represented as exponential and isotropic [116], to represent the nonlinear behaviour of cardiac tissue. Following biaxial experiments in canine myocardium samples [117], transversely isotropic laws were developed [19, 118–123], in order to represent the increased stiffness in the fibre direction compared to the transverse plane. More complex orthotropic laws [33, 115, 124–126] have been developed after the shear data published by [21]. Orthotropic material laws provide a more accurate representation of the passive properties of the ventricular myocardium, and, as shown by [127], transversely isotropic laws can reproduce only three of the six deformation modes from the Dokos dataset. However, more complex orthotropic formulations have more material parameters than simpler transversely isotropic laws, leading to concerns about reliability, uniqueness, and computational cost of parameter identification. For this reason, transversely isotropic material laws are still widely used in clinical applications of mechanics models.

**Figure 4:**
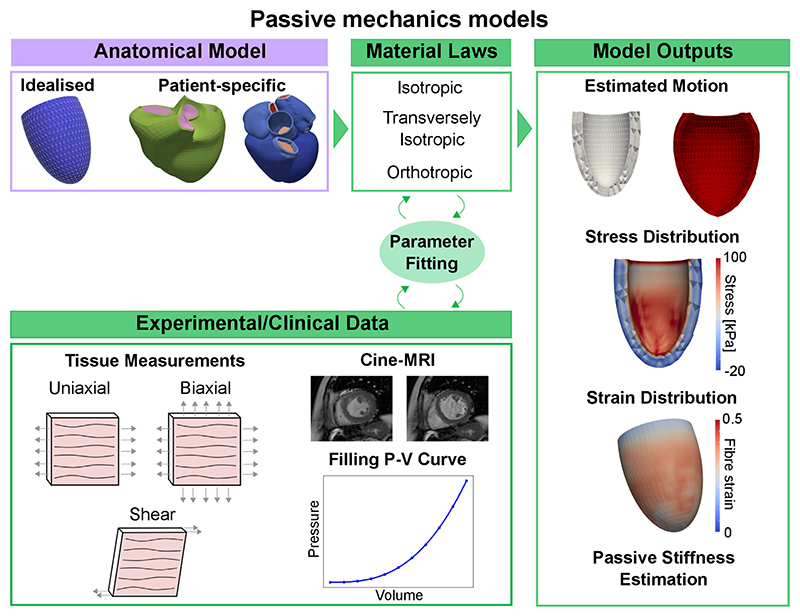
Passive mechanics models pipeline. Using an idealised or patient-specific geometry and a material law representing passive properties of the myocardium, the material parameters can be fitted to match stress-strain relationships derived from tissue measurements, or *in-vivo* cine-MRI data and pressure-volume relationships measured during the diastolic phase. Passive mechanics models can provide an estimation of the patient’s heart diastolic motion, stress and strain distribution and passive stiffness.

Myocardial stiffness provides information about chamber filling and can be used as an indicator for various cardiac pathologies, but it is difficult to quantify *in-vivo*. In this context, cardiac mechanics models can provide a platform to measure passive filling properties of the patient’s heart non-invasively. A typical pipeline to show how anatomical models and material laws are coupled with experimental/clinical data is show in the left side of Figure 4.

This coupling provides an estimation of multiple biomarkers, such as the patient’s heart estimated motion, stress and strain distributions and passive stiffness estimations. We depicted examples on a univentricular mesh in the left of Figure 4. Cardiac mechanics models were first applied to measure passive stiffness *in-vivo* in healthy animals [128–130] and patients [131–134] using cine-MRI data, passive pressure-volume relationships or, alternatively, the Klotz curve. Mojsejenko et al. [135] used diastolic cine-MRI and invasive LV pressure data collected from porcine hearts 1 week after infarction to estimate myocardium and scar stiffness, as well as changes in fibre orientation within the scar. Using cine-MRI patient data, transversely isotropic laws were used to quantify passive stiffness in patients with aortic coarctation and aortic valve stenosis [136], myocardial infarction [137], and to compare the passive stiffness of healthy controls and patients with dilated cardiomyopathy [138], HF [139], and tetralogy of Fallot [140]. Similar works have also been done using 3D printed phantoms [141]. All these applications made use of transversely isotropic laws and, in many cases, the number of estimated parameters was reduced either by using prior relationship between parameters or by fixing a subset of them. This highlights the need for more sophisticated fitting techniques allowing to fit more parameters at a time, or better curated and complete clinical datasets to help constrain a large number of parameters. This will ultimately allow orthotropic material laws to be used in clinical applications.

The material laws and applications described above were designed and performed in the ventricles and were based on experimental data collected from samples of the ventricular myocardium. Only relatively recently, due to the increased interest in atrial dynamics and modelling, existing linear elastic [142], nonlinear isotropic [143] and transversely isotropic [144–146] material laws have been adapted to represent the atrial myocardium. Other groups have proposed atrial-specific and transversely isotropic material laws [147, 148] and fitted the material parameters to the available experimental data from Bellini et al. [22]. Finite element analysis of the atria using a linear elastic model has been used to correlate low-voltage areas and areas with high stresses in the LA in patients with AF [142]. More complex nonlinear, isotropic, and transversely isotropic models have been used to approximate and investigate *in-vivo* stress distribution in porcine biatrial [149] and human LA [147] geometries, showing the potential of atrial modelling to improve our understanding of the mechanical behaviour of the complex anatomy of the atria.

Once active and passive mechanics of the myocardium are represented in a cardiac mechanics model, a representation of the circulatory system can be included to be able to track changes of pressures and volumes inside the cavities. Due to the computational cost, the circulatory system is commonly simulated with lumped parameter models. In the following section, we provide a summary of the state-of-the-art of lumped parameter models to represent the circulatory system.

## 6 Lumped parameter models for the circulatory system

**Figure 5:**
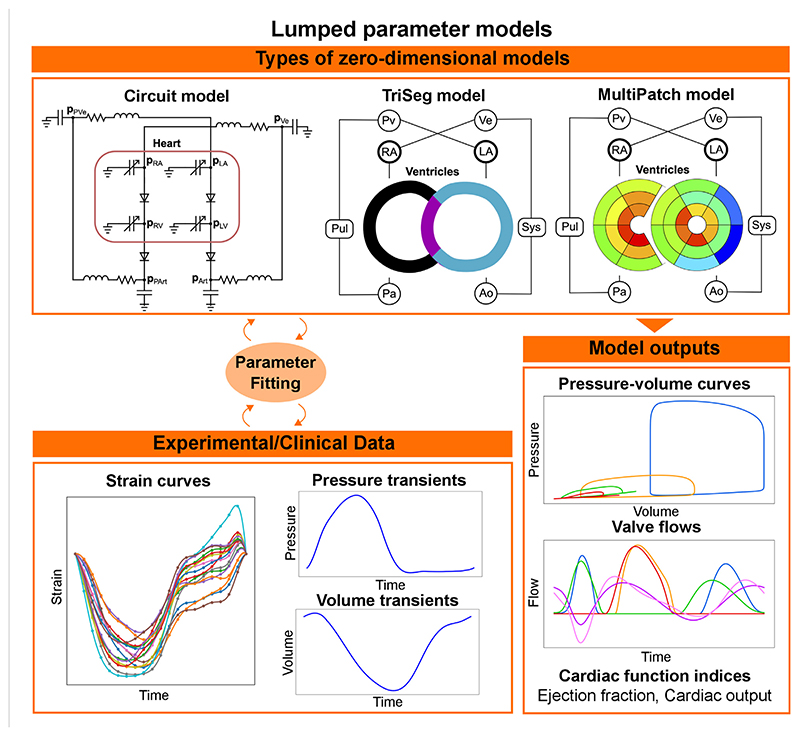
Workflow for lumped parameter models. In lumped parameter models, the circulatory system is modelled as an electric circuit (top left). The cardiac chambers and the other components of the circulatory system are simulated with varying elastance models and a combination of resistance, inductance and capacitance, respectively. To represent local dynamics, the heart can be represented with more sophisticated circulatory system models (TriSeg and MultiPatch). The parameters of the circulatory system can be fitted to available pressure and volume clinical data, or regional strains. Lumped-parameter models are then capable of simulating pressure curves, valve flows and global indices for cardiac function, such as cardiac output and ejection fraction.

Lumped parameter or zero-dimensional models represent the circulatory system as a combination of resistors, capacitors, and inductors, representing the resistance the blood encounters while flowing through different compartments, the energy stored by the vessels’ compliance, and blood inertia, respectively (see Figure 5, top left). The periodic activity of the heart is normally modelled using a varying elastance model. Although lumped parameter models discard spatial information, they constitute an appealing option for clinical applications due to the computational speed, compatible with clinical timescales, and their flexibility, with modules or compartments normally easily replaced by a more complex representation if required by the application of interest. The versatility of lumped parameter models makes them useful in a wide range of applications. Zero-dimensional models of the whole heart, systemic and pulmonary circulations have been applied to investigate diseases such as aortic stenosis and aortic valve regurgitation; and to simulate pulmonary hypertension to better understand the underlying causes of the disease and improve treatment [150]. They have also been used to test, using simulations, different types of ventricular assisted devices in peadiatric [151] and adult patients with HF [152]. An even simpler setup, including only the left side of the heart and the circulatory system, has been used to match the pressure-volume relationships measured from different stages of HF, in order to provide a visual representation of the stages of HF from the American Heart Association [153]. An example of these pressure-volume curves can be seen in the bottom of Figure 5. Other indices such as ejection fraction, cardiac output or flow transients can be obtained from such circulatory system models, see bottom right of Figure 5.

Although lumped parameter models can be used for clinical applications because of their low computational demand and flexibility, some of their parameters do not necessarily have a physiological meaning, which makes it difficult to estimate their values from clinical or experimental data. To improve parameter identifiability and reduce the number of unknown parameters, Arts et al. developed CircAdapt [154], a lumped parameter model for the heart and circulatory system that includes adaptation rules capable of updating geometry parameters such as the wall volumes of the chambers and the wall areas of the vessels depending on the load they experience. Subsequent developments of CircAdapt were able to account for the mechanical interaction between the LV free wall, the right ventricle (RV) free wall and the septum (TriSeg model) [155], and for heterogeneity of cardiac tissue properties (MultiPatch model) [156], see top of Figure 5. In contrast to other lumped parameter models, which discard spatial information, these versions of CircAdapt provide local as well as global dynamics of the heart. This makes CircAdapt suitable for studying diseases or treatments in which LV-RV interaction and/or spatial heterogeneity are important. The TriSeg model has been used in combination with echocardiography strain data to investigate different substrates in RV arrhythmogenesis cardiomyopathy, and was able to link RV deformation abnormalities to changes in RV contractility and compliance [157, 158], to identify potential causes of abnormal septal motion in patients with pulmonary hypertension [159] and to link cellular contractile properties to CRT response [160]. The MultiPatch model has been used to investigate local work heterogeneity during different pacing modalities to compare various CRT delivery methods [161]. Depending on the application, CircAdapt and its extensions constitute a valuable tool for *in silico* investigations. Furthermore, thanks to its modular structure, CircAdapt can also be coupled with three-dimensional mechanics models to have a more sophisticated representation of the electromechanical function of the heart. In the following section, we provide a summary of the state-of-the-art of tissue- and organ-level cardiac electromechanics modelling, showing their integration with active and passive mechanics and the incorporation of lumped parameter models.

## 7 Three-dimensional electromechanics models

**Figure 6:**
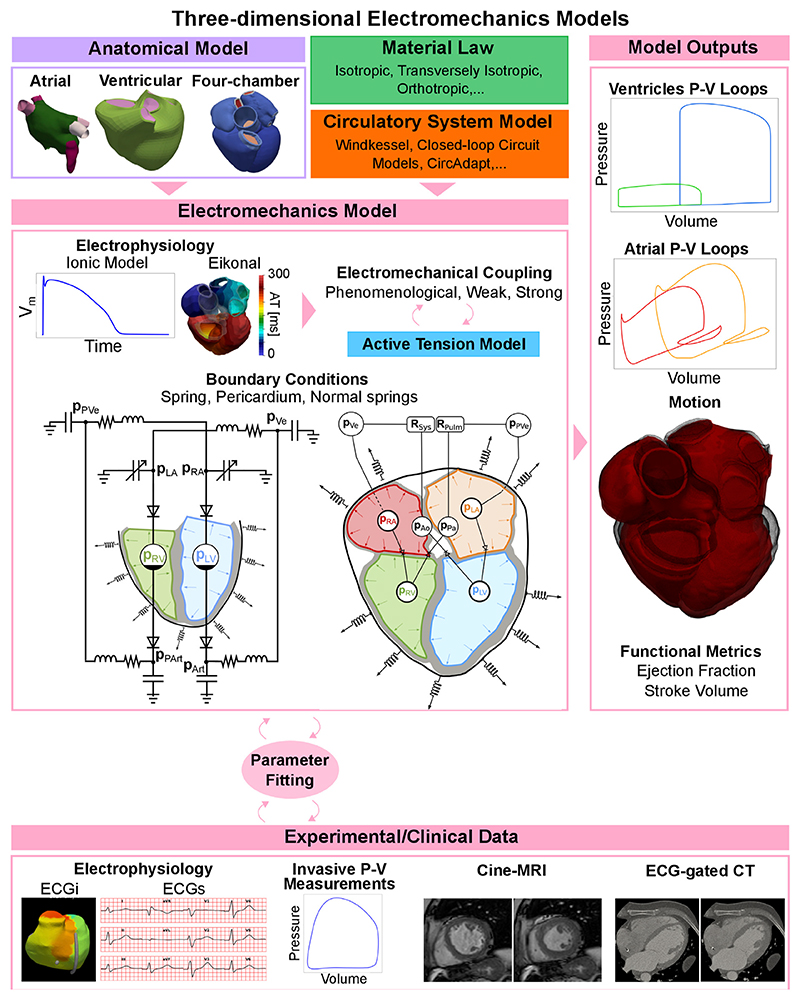
Three-dimensional electromechanics models pipeline. Depending on the application, tissue-level mechanics models can include to one chamber, two chambers, or the whole heart. A material law and a circulatory system model are needed to represent passive behaviour of the myocardium and the preload and the afterload of the chambers. The electrophysiology model is coupled with an active tension model to simulate active contraction, and the parameters are fitted to available clinical data. The electromechanics model can output pressure-volume loops for the chamber(s) of interest, the motion of the anatomy and functional metrics such as ejection fraction and stroke volume.

In order to simulate the electromechanical activation of the heart at the tissue level, an electrophysiology simulation is often needed, see Figure 6, top-centre. Before focusing on three-dimensional electromechanics models, we briefly explain electrophysiology simulations, as these are an essential building block of an electromechanics model. In essence, an ionic model is coupled with a tissue-level electrophysiology model and computes transmembrane potentials and/or other variables of interest, such as cytosolic calcium concentration. These variables (activation times, transmembrane potential, etc.) are then passed to the active stress model to trigger active tension development, represented with an active cell mechanics model. Electrophysiology and mechanics models can be coupled in multiple ways. We talk about phenomenological coupling if the tension development in the cell model is triggered with an activation time, discarding the cross-bridge cycle and the calcium transient. A detailed ionic model can otherwise provide a calcium transient that can be passed to the cell model (weak coupling). Both phenomenological and weak coupling neglect the effect of mechanical deformation on electrophysiology. Strong electromechanical coupling instead accounts for bidirectional coupling through stretch-activated channels and length-dependence calcium buffering of troponin C [162]. Although strong coupling is often not used due to its high computational cost and complexity, accounting for such mechanisms can be important for some applications, such as arrhythmias, where stretch-induced activation can serve as a trigger for ectopic beats. A review of the different ionic and tissue-level electrophysiology models is beyond the present scope. For more information, we refer the reader to [163].

In Figure 6 we show a schematic representation of all the different components of three-dimensional electromechanics models. The electrophysiology output, together with boundary conditions such as springs, the pericardium, or normal springs constrain the mechanics model, both at the initial step of the simulation (initial conditions) and at each timestep (boundary conditions), as shown in the centre of Figure 6. Multiple outputs such as pressure and volume curves, motion, ejection fraction or stroke volume are produced, see left column of Figure 6. These results can be fitted to experimental or clinical data for validation (Figure 6, bottom row). Depending on the application, tissue-level mechanics models can include one chamber, two chambers (usually the ventricles or atria), or the whole heart. In this section we review the research done with electromechanics simulations, depending on whether they are done with ventricular, atrial, or four-chamber models. We use the terminology ‘tissue-level’ here to denote cases when not the whole organ is modelled, but only specific chambers. We refer to ‘tissue-level’ simulations to denote studies where only specific chambers rather than the whole heart was modelled.

### 7.1 Ventricular models

Ventricular mechanics models were the first cardiac models to be formulated at the tissue level mainly due to data availability and geometric simplicity. As early as 1892, Woods applied Laplace’s theorem to the LV assuming it to be a sphere [164]. Since then, multiple ventricular models have been used, from truncated ellipsoids to patient-specific anatomies.

Simplified ventricular geometries have been used to link subcellular simulations with simulation of the entire chamber [105] in patients with dilated and hypertrophic cardiomyopathies. Despite using a simplified LV geometry, this approach allows to link protein unction to observable clinical biomarkers after administering antiarrhymtic drugs, using a Huxley-based cell model. This simplification of the ventricular geometry makes it more manageable to investigate how changes at the subcellular level, such as the effects of drugs or alterations in proteins, influence the behaviour at the organ scale. Similarly, to translate mechanical behaviour from the cellular to the anatomical level, in [165, 166] the authors coupled the mechanical model of MyoSim to a simple hemispherical representation of the LV. In [100], Gusseva et al. combined a hybrid Hill-Huxley model with a spherical representation of the RV in a cohort of Tetralogy of Fallot patients after pulmonary valve replacement, to assess the predictive value of the mechanics models before and after replacement using a transversely isotropic material law.

Biomechanics ventricular models have been widely used to study therapies such as CRT. In [55], Sermesant et al. simulated the transversely isotropic mechanical behaviour of a biventricular mesh from a patient undergoing CRT using MRI and pressure catheters. In [53], Niederer et al. formulated a new active tension law in combination with a transversely isotropic material law to assess the influence of length dependence on failing hearts undergoing CRT. This law was later used by the same group to assess the distribution of work in CRT patients [56]. In [56], the authors used biventricular mechanics simulations to find the optimal location of the LV pacing lead based on the acute hemodynamic response. Gerach et al. [167] performed a CRT study using simplified LV geometries in a cohort of patients. These were combined with patient-specific electrophysiological measurements to quantify mechanical torsion in HF patients.

Mechanics models can be used to integrate electrophysiology models, which might lack sufficient data for certain diseases or applications, and computational fluid dynamics models, which may have demanding technical needs and high computational expenses. For example, multiple mechanics models have been used to study how physiological and pathological electrophysiology are impacted by cardiac mechanics. In [168], Salvador et al. modelled LV tachycardia using electromechanic simulations. They used a patient-specific geometry, scar location, pressure, and volume biomarkers. One of the main findings was that, due to the mechanoelectric feedback, tissue deformation affects the activation pattern of ventricular tachycardia. The authors extended this study in [169] to investigate the role of stretch-activated channels on the arrhythmia dynamics, finding that tissue deformation could affect the basic cycle length of tachycardia but not its stability. A transversely isotropic law and a Hill-based cellular model were used. The role of the mechanoelectric feedback was previously studied by Hermeling et al. [170], where, using lumped parameter models, Huxley-based cell models, and a simplified geometry, the authors found that the mechanoelectric feedback could partially explain electrophysiological characteristics such as T-wave memory in ventricular pacing. Finally, in [171] Adeniran et al. studied the effect of adding stretch-activated channels to ventricular models using a Huxley-based cell model, finding that it can be an essential feature to adequately model short QT syndrome.

Studies where a computational fluid dynamics model might have been adequate but was too prohibitive due to computational cost and complexity have also used ventricular mechanics models. This will depend on the context, as in some cases analysing the motion, pressures or stress, for instance, is enough and the specific blood flow patterns are not needed. In [172], Bakir et al. simulated ventricular assist devices for patients with dilated cardiomyopathy on idealised biventricular meshes, combining a simplified biventricular geometry and using an orthotropic material law with high-fidelity blood flow simulation in the cavities. This device was also modelled by Sack et al. in [173], where the authors focused on the effects of the device on septum deformation. An orthotropic material law was also used with a Hill-type active tension model. In [54], the authors compared biomarkers such as myofibre stress and strain in a patient with pulmonary hypertension with a healthy control using personalised biventricular models anatomically (from MRI) and functionally (through pressure catheters), while using a transversely isotropic material law. Therapies for complex and rare diseases such as Tetralogy of Fallot were modelled in [174], where Tang et al. simulated patch modelling for RV reconstruction comparing stress and strain distributions for different transversely isotropic patch designs [174] using delayed enhancement MRI to locate the patches and valves. In [175], Cutrì et al. modelled the ventricle of a patient with hypoplastic left heart syndrome. The authors used an orthotropic material law and a Hill-based cell model and simulated the effects of surgery.

Alternatively, ventricular mechanics models have also been used to achieve clinical translation mainly through two ways: conducting *in silico* trials and reducing the burden of expensive and not readily available imaging technologies such as CT and MRI. We use the definition of *in silico* trial used before in [176]: a research study that uses computer models of cells, tissues, organs, or systems of human subjects, assigned to one or more interventions (which may include some form of control group) to evaluate the effects of those interventions on health-related biomedical or behavioural outcomes. In [109] Margara et al. performed a set of *in silico* trials using ventricular mechanics to investigate the effect of specific mutations in hypertrophic cardiomyopathy. This setup was based on the one presented by Wang et al. [177]. In [177], Wang et al. presented a framework to simulate electromechanic simulations on a patient-specific biventricular mesh. Parameters were chosen from the literature or tuned to achieve physiological pressures and volumes, and a Hill-based model for active tension and an orthotropic material law were used. The purpose was to demonstrate the feasibility of such a framework. The clinical availability of imaging technologies can heavily influence the development of anatomical and functional models. In [178], Krishnamurthy et al. presented a pipeline that combines multimodal data (CT, catheter data, echocardiography) and integrates them into closed-loop biventricular mechanical simulations using a Hill-type cell model in patients with HF. Instead of using CT or MRI images, Aguado-Sierra et al. [179] created biventricular meshes from echocardiography images and ran mechanics simulations on HF patients using an orthotropic material law. Although this technique has lower resolution than CT or MRI, it might allow greater translation in environments where costly scans are not available. Additionally, models can be used to reduce the imaging burden using more accessible and readily available imaging technologies to provide insights into the patient’s heart, that otherwise would require more complex and time-consuming image acquisition protocols. In [180], Peña et al. also used echocardiography to create ventricular models but in foetuses at different stages and assess which properties (such as active tension) change over time. The authors used a transversely isotropic material law with a Hill-based active tension model.

### 7.2 Atrial models

The development of three-dimensional atrial models has lagged behind that of ventricular models, as the atria were long believed to contribute little to overall heart function. However, in recent years, the importance of atrial function and its role as an indicator of cardiac health have been increasingly recognised, triggering the development of atrial models. Typically, three-dimensional atrial models fall into two types: LA-only models and biatrial models, including both LA and RA. In terms of clinical applications, most atrial models have been used to investigate AF and the response to catheter ablation procedures. However, the vast majority of these atrial models focus only on atrial electrophysiology and neglect biomechanics.

Although the number of existing atrial biomechanics models in the literature is limited, they can have significant clinical value. Adeniran et al. [144] used a multiscale, transversely isotropic biatrial model to investigate how electrical remodelling induced by persistent AF affected atrial mechanics and leads to loss of atrial contraction commonly associated with persistent AF. A remodelled state was created by modifying a family of electromechanically coupled single cell models, previously validated against the F-Ca relationship of human atrial myocytes [181]. It has also been possible to create an AF pathological state in atrial models through tissue- and organ-level modifications, without considering cellular remodelling. Feng et al. created an AF state in their LA-mitral valve model [182], without considering cell-level characteristics, but simply by removing atrial contraction. Feng’s atrial model of healthy individuals included a transversely isotropic material model and used a phenomenological approach with a simplified active stress to model active contraction. In the AF case, the active stress was set to zero and represented only an advanced AF case where the atrial booster function is completely lost.

Given the number of remodelling mechanisms associated with AF, mechanics models can benefit from including more information. In the atrial model by Moyer et al. [183], the authors included information about changes in material properties, size, shape, pressure, and conduction. The authors used a transversely isotropic LA model with a Huxley-based cell model coupled to a hydraulic circuit model of the LV and pulmonary circulation to examine how AF-related remodelling could affect mechanics [183]. In this study, the baseline configuration was created from the average MRI-measured geometry of a cohort of healthy patients and loaded using scaled pressure-time curves measured from patients with paroxysmal AF prior to an ablation procedure. The mechanics model incorporated the contact between the LA and surrounding structures by applying inward pressures to the LA septum, anterior wall, and roof. Furthermore, a representation of the LV contraction force acting on the LA was included by applying a downward force driven by the displacement of the mitral valve measured along the pulmonary vein-mitral valve axis of the patient’s MRI. The baseline atrial model was modified to investigate the effect of common factors related to AF on atrial function. Modified factors included size - changing the geometry of the anatomical model so that the end-diastolic endocardial volume matched the average value measured from a cohort of AF patients; shape–modifying the geometry of the anatomical model to match the average geometry measured from patients with AF but scaled to match healthy atrial volumes; pressure - increasing the measured healthy pressure load curves; and fibrosis burden - increasing the isotropic stiffness terms in the myocardium material model. With pathological state atrial models, the global LA function could be examined with reference to the healthy case, illustrating the effect of the AF-related factor using simulated pressure-volume loops.

The baseline Moyer et al. atrial model [183] was further built upon in the paper by Phung et al., where three common scar tissue patterns created during catheter ablation procedures were added, to investigate the effect of scar on mechanical function [146]. Here, scar tissue was modelled in the atrium for the first time by stiffening the isotropic material parameter in the myocardial material model to an even greater extent compared to the increase made when modelling fibrosis, along with the elimination of anisotropy and active contraction [146]. Regional and global functions were assessed using the regional motion of the atrial wall segments and the pressure-volume loops, respectively. With these functional metrics, the effect of each ablation pattern on atrial function was examined with respect to the baseline atrial model. Here, the authors highlight the value of this type of investigation in planning and guiding ablation strategies to correct arrhythmia while having minimal effect on normal atrial function. The pressure-volume loops generated with this mechanics model, however, did not reproduce the characteristic figure-8 shape observed clinically. This could be due to issues surrounding the boundary conditions applied to the LA or to the tuning of the lumped parameter model used to represent the LV and pulmonary circulation. Although the characteristic atrial pressure-volume loop is difficult to achieve *in silico*, the simulated pressure-volume loops still provide valuable information on how the atrial function is affected under various conditions.

Atrial model output, including simulated motion, volume, and pressure data, is rarely validated in the literature (only in [183], Moyer et al. validated their mechanics model), due to lack of patient data. Additionally, the lack of patient data affects the construction of mechanical models of the atria. Many atrial models assume a constant pressure based on literature values [142, 144] due to difficulty in obtaining patient pressure trace data. Another issue related to atrial model construction is that none of the existing human atrial models mentioned the incorporation of a pericardium. However, the importance of the pericardium for cardiac biomechanics has recently been highlighted by computational studies [62, 184, 185]. Limitations of existing imaging methods have also affected the construction of atrial models. In the literature, many atrial models assume a uniform wall thickness [142, 144, 146], while physiologically, the atria have a heterogeneous wall thickness, ranging between 0.5 and 6.5 mm [186–188]. This limitation persists because the thin walls of the atria require high-resolution imaging to be captured. However, the study by Feng et al. [182] showed a difference in mechanical behaviour when a uniform wall thickness was assumed compared to a patient-specific wall thickness. In a study by Augustin et al. [147], the authors suggested a correlation between heterogeneous wall thickness and LA mechanical behaviour. As such, in the future, to derive the most clinical realism, it may be necessary to include patient-specific wall thickness in 3D atrial models when atrial mechanics is of interest. It should be mentioned, however, that the thin walls of the atria present some difficulties with respect to stability and convergence of the mechanical solver. Augustin et al. highlighted challenges in estimating the unloaded configuration [147] of the atria due to their thin walls. Simulated pressures might then be limited to values lower than physiological ranges, or myocardial stiffness may be increased in an attempt to avoid over-inflating the atria [147].

Atrial mechanics models are in their infancy compared to ventricular models and, as highlighted above, present significant challenges. However, due to the now widely recognised role that atria play in overall cardiac function, atrial models are rapidly developing, alongside novel atrial-specific imaging and functional data collection methods to capture the thin atrial walls and to better understand atrial physiology. The integration of richer clinical datasets from the atria into mechanics models will make atrial computational mechanical models more suitable for clinical applications.

### 7.3 Four-chamber models

Electromechanical models of the whole heart are increasingly being used to provide mechanistic insight into cardiac pathologies and predict clinical output metrics on the organ scale [189–192]. Although they suffer from higher computational cost and stability issues, four-chamber models imply significant advantages compared to biventricular models: they avoid the need for an unphysiological spatial boundary condition on the base or apex of a biventricular mesh, and incorporating the atria in combination with a closed-loop circulatory system also provides physiological preload and afterload to the ventricles. In addition to improved boundary conditions, four-chamber models are able to capture the complex atrioventricular interaction, which are critical in the study of cardiac pathologies affecting the whole organ. Despite the relative novelty of four-chamber biomechanics, there have already been successful applications for investigating pharmaceutical effects [189], evaluating surgical treatment strategies [190], and questioning the validity of computationally-derived clinical metrics [191].

Some applications, traditionally investigated through electrophysiological studies only, would benefit from the inclusion of biomechanics. An example of this is the use of four-chamber models to investigate cardiac arrhythmias. Peirlinck et al. [189] used a four-chamber electromechanical model coupled to a closed-loop circulatory system to investigate the proarrhythmic potential of pharmaceutical therapy. The mechanics model used a transversely isotropic hyperelastic material and implemented a Huxley-based law for the active stress modelling, including the active stress interaction between adjacent muscle fibres along the sheet direction. By blocking the pharmacologically affected ion channels, the mechanics model was used to link the effect of antiarrhythmic drugs on a single ion channel to the effect on the whole heart and system function.

The clinical application of four-chamber modelling to surgical planning for cardiac arrhythmias is also becoming increasingly feasible. Electromechanical models generated from healthy patients have been modified to include radiofrequency ablation scars typical of a surgical treatment strategy for AF [193]. Gerach et al. developed a four-chamber model using an orthotropic material law and a Huxley-based model for active tension. This model was embedded in an explicit representation of the pericardium, as in [185] and coupled to a closed-loop representation of the circulatory system. By defining the scar regions as non-conducting and simulating multiple heart beats, Gerach et al. found that the efficiency of ventricular pumping was not significantly affected by the atrial scar. This work was extended to include the increased mechanical stiffness of scar tissue and the new configuration was used to analyse combinations of five commonly used ablation scar patterns [190]. The authors concluded that the position and extent of ablation scars have implications not only for atrial pressures and stroke volumes but also for ventricular performance. These *in silico* experiments represent a promising step towards using four-chamber heart models to optimise choice of ablation strategy in a specific patient.

The four-chamber model of [193] was also extended by embedding the heart in a torso to investigate the influence of contraction on ECG [191]. With a conclusion similar to that from [194] but in a four-chamber heart, the model suggested that some features of the T-wave are significantly affected by cardiac motion. This has implications for the clinical translation of purely electrophysiological models, as some ECG features may not be accurately predicted without a full electromechanical approach.

Whole-heart models have also been used to study myocardial infarction [192]. Jafari et al. developed a four-chamber model using a transversely isotropic, hyperelastic material law for passive mechanics and implemented the active mechanics using a Huxley-based model accounting for sarcomere length and mechanical activation. This model was coupled to a lumped parameter representation of the circulatory system. An infarcted region was defined in the LV wall and the contractility of this region was reduced to simulate acute tissue damage after MI. A comparison of cardiac motion and pressure-volume loops for the healthy and infarcted heart indicated a reduction in cardiac work immediately after MI. The use of a four-chamber geometry removes the need for nonphysiological boundary conditions that require extensive parameter tuning to match the simulated motion with data acquired from the participant.

Four-chamber electromechanical models have also been used to investigate cardiac remodelling and its effect on cardiac function. Strocchi et al. [195] varied the orientation of the ventricular fibres and studied the effect on the displacement of the atrioventricular plane, atrial pressures, and venous return. Land et al. [196] used a four-chamber model to investigate AF-induced electrophysiological remodelling of the atria considering changes in calcium dynamics after restoration of sinus rhythm. The authors concluded that remodelling led to a lower peak atrial pressure and reduced atrial relaxation, as well as a small reduction in ventricular filling. Finally, Genet et al. created a four-chamber model for cardiac growth and remodelling during chronic HF by simulating parallel and serial deposition of sarcomere [197]. In addition to predicting wall thickness, chamber volume, and cardiac geometry, the four-chamber model can predict papillary muscle dislocation, annular dilation, and other clinically relevant outcomes. This work highlights the potential four-chamber models have for predicting the progression of HF in specific patients and promises to aid in personalised treatment planning for HF in the future.

## 8 Machine learning to reduce computational burden

One of the main barriers to the clinical translation of cardiac biomechanics models is the computational cost, as simulations usually require several hours of hundreds of cores in high-performance computing facilities [198].

Recently, a new approach has been presented to accelerate ventricular mechanics simulations: combining them with neural networks to reduce the computational burden. In [199] Motiwale et al. tested this approach with an idealised LV represented as a truncated ellipsoid. The neural network was trained by minimising certain residual force vectors obtained from the weak form of the partial differential equations that drive a simulation, achieving a speedup of 4 orders of magnitude.

Two of the main bottlenecks when running cardiac mechanics models are in parameter fitting and in sensitivity analysis. In both cases, multiple combinations of parameters must be chosen to run simulations and analyse the output. Although the different techniques of parameter fitting and sensitivity analysis are beyond the scope of this review, machine learning techniques such as Gaussian process emulators are being used to alleviate the computational cost [200]. In [200], Strocchi et al. ran multiple mechanics simulations to then train a set of Gaussian process emulators. These emulators were then used to rapidly and finely sample the parameter space and find the most influential simulation parameters. Similarly, in [201], Salvador et al. used latent neural ordinary differential equations to perform a global sensitivity analysis and parameter estimation, learning not scalar values, but temporal traces. Trained with 400 multiscale mechanics simulations, this approach allowed for simulations 300 times faster than real time on a single processor of a standard laptop.

## 9 Discussion

One of the end-goals of cardiac biomechanics models is clinical translation: to improve the clinical decision-making process, to improve and/or optimise a therapy outcome, and to gain more knowledge about a disease. Although this field is not new, there is still a gap before biomechanics models can fulfil this purpose.

In most of the papers discussed in this review that describe tissue-level simulations, only a few virtual patients were analysed (*n* < 20). Although a few patients might suffice for a pilot study, if we want to make general statements that will be true throughout the wider population, more virtual patients are needed. The main reasons for this shortcoming are 1) the time-consuming, expert-requiring pipelines to generate patient-specific anatomical models from imaging data and 2) the computational cost of simulations. For the former problem, reliable, validated, up-to-date and ideally open-source software tools are needed to accelerate simulation pipelines. For the latter, machine learning-based solutions can be used to reduce computational cost.

### 9.1 Enabling clinical translation through simulation software

At present, there are multiple software platforms to simulate cardiac mechanics, possibly due to the complexity of the systems considered, their multiscale and multiphysics nature, and the ever-evolving field in terms of solvers, physiological findings, and user-specific needs.

Cell models, such as the one used in [98], can be run in the MyoSim (or MATMyoSim) software^1^. MyoSim, able to simulate half or full sarcomeres, monitors the distribution of cross-bridge strains at individual binding sites over time, dynamically adjusting these distributions as the cross-bridges undergo the power stroke or as the filaments slide past one another. This explicit representation of cross-bridge strain, while increasing the computational load relative to the mean-distortion approach, allows for a finer characterisation of strain-related effects, including cooperativity between near-neighbouring cross-bridges. MyoSim simulations have been included in finite-element simulations [202]. The FiberSim^2^ platform, developed by the Campbell Muscle Lab, extends the MyoSim modelling approach to provide more flexibility to simulate spatially explicit phenomena [103]. Another option for cell simulations is MUSICO^3^ [104], a simulator developed by the Mijailovich Lab, based on Monte-Carlo modelling. Although not explicit, this approach exploits experimental measurements to predict contraction properties via sarcomere-level regulation pathways.

In some cases, simulation software platforms that initially focused purely on electrophysiology have broadened their scope to include mechanics, for example, the Cancer, Heart and Soft Tissue Environment (CHASTE)^4^ and the Cardiac Arrhythmia Research Package (CARP)^5^. In both cases, a cell dynamics-electrophysiology-mechanics coupling is possible from the microscale to the macroscale, although the electrophysiology is more established, with Cardiac CHASTE^6^ and OpenCARP^7^, respectively, compared to mechanics.

Some research groups have followed a top-to-bottom approach for software development. Instead of developing a tool specifically for cardiac mechanics simulations, general-purpose software (usually finite element-based) has been used for cardiac simulations. Some examples include SimCardEMS^8^, from the Simula Laboratory based on FEniCS, Ansys^9^, FEBio^10^ or Abaqus^11^, from Dassault Systemes.

To allow clinical translation, thorough benchmark tests should be performed on the simulator, serving as a verification step before focussing on specific applications. Benchmark studies such as the one provided by Land et al. [203] are essential to achieve this goal. These recommendations were also described in the Food and Drug Administration (FDA) draft on the credibility of simulations in medical device submissions [204]. Although focused on medical devices and not encompassing only mechanics modelling, these recommendations are easily applicable to facilitate clinical translation of cardiac biomechanics models.

### 9.2 Learning from the existing regulatory framework

Clinical decisions are traditionally built on evidence from bench, animal studies, and clinical trials. All this information is usually collected in guidelines and recommendation guides. Recently, some guidelines have contained digital evidence, either from *in silico* studies or even *in silico* clinical trials. One of the most recent is the FDA draft on credibility for simulation and modelling of clinical devices [204], built on their previous report from 2016 [205]. Even if a study is not intended for an FDA submission of a medical device, practises such as code verification, validation, and uncertainty quantification are necessary steps to increase trust in mechanics models. However, these steps are rarely performed, even in *in silico* clinical trials [176].

Although lacking specific regulatory guidelines for best practise, mechanics modelling studies are already being used to support regulatory submissions at different stages [206], allowing them to complement clinical trials in aspects such as predicting performance beyond instructions for use, cost and time. In the United Kingdom, a recent report highlighted success stories of modelling and simulations in healthcare [207]. However, these cases do not yet include cardiac biomechanics, but cardiac electrophysiology or noncardiac biomechanics.

### 9.3 The gap between experimental data and models

The challenge of reliably calibrating and validating simulations with experimental data also increases with model complexity. For example, an important source of experimental data for these purposes is derived from skinned muscle preparations. This experimental system allows for a more direct characterisation and control of sarcomere biomechanical reactions than can be inferred from intact cells. However, the interpretation of these measurements in the context of intact cells faces significant challenges [25]. To a large extent, the interpretation of the measurements themselves often necessarily rests on model-based assumptions. Variations in measurements can be the result of experimental uncertainty, but also variability inherent in muscle samples. Unless addressed, these challenges will only increase in the future with novel techniques, such as experiments with engineered heart tissue [208]. Consequently, the expectation of achieving an ultimate model that is suitable for all purposes might be unreasonable. Therefore, the specific context of use should be established beforehand, as recommended by the FDA [204].

The lack of biophysical mechanisms of cell-scale contraction has yet to be fully clarified. Previously unconsidered phenomena are being established, for example, the tension-dependent regulation of thick-filament activation involving the myosin “off” state. Another issue that needs further investigation is that of tissue heterogeneity in cellular properties within a tissue [209–211]. This effect, unrepresented by the assumption of homogeneity in most existing models, can affect simulated cardiac muscle performance [212, 213].

On the experimental side, novel findings, including tissue staining [214] and proteomics [215] can provide novel tools to characterise tissue heterogeneity and find changes in response to disease. In [215], Linscheid et al. provided the largest dataset of cardiac protein expression from human samples collected *in-vivo*, classified by chamber-specific expression. Cardiac conexins (a class of proteins) have also been linked to specific tissue, for example, conexins Cx43 are expressed mainly in atrial and ventricular myocytes and less expressed in the conduction system such as Purkinje fibres, where conexins such as Cx40 are more ubiquitous [214]. These findings provide a potential bridge between the microstructure and the whole-organ level. However, it can be difficult to identify how specific changes in proteins affect cardiac function *in-vivo* or in bench studies. Multiscale modelling can bridge this gap using techniques such as global sensitivity analyses [200].

### 9.4 Future directions

Cardiac anatomy is highly variable depending on sex, subject size, and from healthy to diseased state, and adjusting for this variability is often important. Cardiac atlases built from a collection of patient-specific anatomical and mechanics models provide a statistical representation of the anatomy of a cohort. Multiple datasets of CT and MRI have been used to create biventricular [216–218], LA [81], biatrial [219], and whole heart atlases [198]. Statistical shape models have been used to investigate the LV shape induced by preterm birth in adults [220], obesity in adults [221] and children [222]; to identify potentially favourable remodelling as a response to CRT in patients with HF [223] and to quantify LA anatomical remodelling in patients with AF [224]. Although complex and computationally demanding, accounting for shape variability in cardiac mechanics models would make *in silico* mechanics models more relevant for clinical applications.

A different approach to account for patient intervariability would be to include genomic information. Currently, mechanics models include genetic effects mainly in the shape of expressed proteins, for example, with changes in ion channel properties. Including the vast amount of genetic information that can now be collected routinely would improve the personalisation level of patient-specific cardiac mechanics models.

Although multiple cardiac mechanics models used are already multiscale, there are still several relevant processes that are not being routinely included in these studies, namely, the link to other organs, perfusion, and energetics. In this regard, the recent work by Sharifi et al. [225] is notable, where the authors were able to mimic important features of the physiological baroreflex, one of the body’s homeostatic mechanisms that helps to maintain blood pressure at nearly constant levels. Regarding perfusion, the process linking the circulatory system with the myocardium, Zingaro et al. [226] recently presented a mechanics model linking cardiac mechanics with perfusion. However, this is just the first step to achieve clinical translation of this type of cardiac model. Lastly, modelling energetics can be useful, for instance, in some cases of HF, where there are abnormalities in how heart cells produce energy. Works such as that by Randall et al. [227] show how to model cardiac energetics, but there is a missing link with whole-organ cardiac mechanics. With all these advances, we could better model representations of physiological conditions, including exercise.

Models of cardiac growth and remodelling focusses on how heart structure changes after different stimuli. Changes are usually driven by a mechanical stimulus such as a pressure overload. This type of modelling is not routinely integrated with biomechanics modelling, due to several reasons. Firstly, the interplay between the growth law and the mechanics simulation is not clearly established. The meshes where growth and remodelling models are tested are mainly ventricular or biventricular and mostly very regular or ideal ellipsoids. This simplification hinders clinical translation and integration with patient-specific biomechanics models. Lastly, multiple growth laws are formulated with different advantages and disadvantages. Although it is unlikely (as happened with the passive material laws) that a single law becomes the only one used, more research is needed to understand the limitations of the available growth laws and the implications in the study, potentially requiring integration with clinical and/or experimental data. For a review of the existing growth and remodelling cardiac models, we refer the reader to [228]. These models would also benefit from adopting a multiscale approach, linking down to subcellular growth and remodelling models similar to those used in system modelling [229].

## 10 Conclusion

The clinical translation of cardiac biomechanics models is essential to have a societal impact by improving the lives of patients. Cardiac biomechanics models provide an excellent framework for integration into the clinical pipeline, but more refinement is needed. In particular, the context of use must be adequate to the high computational cost of biomechanics models, with a focus on better validation against clinical data.

## Footnotes

1
https://campbell-muscle-lab.github.io/MATMyoSim/

2
https://campbell-muscle-lab.github.io/FiberSim/

3
https://www.solindies.com/musico

4
https://www.cs.ox.ac.uk/chaste/

5
https://carpentry.medunigraz.at/

6
https://www.cs.ox.ac.uk/chaste/cardiac_index.html

7
https://opencarp.org/

8
https://computationalphysiology.github.io/simcardems/

9
https://www.ansys.com/en-gb/applications/cardiovascular

10
https://febio.org/knowledgebase/case-studies/structural-mechanics/cardiac-mechanics-benchmark-problems/

11
https://www.3ds.com/products-services/simulia/products/abaqus/
